# Effectiveness of Implantation of Cardioverter-Defibrillators Therapy in Patients with Non-Ischemic Heart Failure: an Updated Systematic Review and Meta-Analysis

**DOI:** 10.21470/1678-9741-2017-0003

**Published:** 2017

**Authors:** Zhenhua Xing, Liang Tang, Chenyang Chen, Jiabing Huang, Zhaowei Zhu, Xinqun Hu

**Affiliations:** 1 Second Xiangya Hospital of Central South University, Department of Cardiology, Huan Province, P.R. China.

**Keywords:** Defibrillators, Implantable, Heart Failure, Cardiomyopathies/*Therapy, Meta-Analysis

## Abstract

**Objective:**

Implantable cardioverter-defibrillator has become the first-line therapy for prevention of sudden cardiac death. Controversial results still exist regarding the effectiveness of implantable cardioverter-defibrillator (ICD) in non-ischemic heart failure.

**Methods:**

The PubMed, Embase, and Cochrane Central databases were searched for randomized trials comparing implantable cardioverter-defibrillator in combination with medical treatment *versus* medical treatment for non-ischemic heart failure. The primary endpoint was incidence of all-cause death. We derived pooled risk ratios with fixed-effects models.

**Results:**

Five studies enrolling 2573 patients were included. Compared with medical treatment, implantable cardioverter-defibrillator with medical treatment was associated with a significantly lower risk for all-cause mortality (Risk ratio: 0.83; 95% confidence interval 0.71 to 0.97).

**Conclusion:**

Compared with medical treatment only, implantable cardioverter-defibrillator in combination with medical treatment reduces all-cause mortality.

**Table:** 

Abbreviations, acronyms & symbols				
AMIOVIRT	= Amiodarone versus Implantable Defibrillator Randomized Trial		DEFINITE	= Defibrillators in Non-Ischemic Cardiomyopathy Treatment Evaluation
CAT	= Cardiomyopathy trial		ICD	= Implantable cardioverter-defibrillator
CI	= Confidence interval		LVEF	= Left ventricular ejection fraction
COMPANION	= Comparison of Medical Therapy, Pacing, and Defibrillation in Heart Failure		NYHA	= New York Heart Association
CRT	= Cardiac resynchronization therapy		RCTs	= Randomized clinical trials
CRTD	= Cardiac resynchronization therapy-cardioverter–defibrillator		RRs	= Risk ratios
CT	= Computed tomographic		SCD	= Sudden cardiac death
			SCD-HeFT	= Sudden Cardiac Death in Heart Failure Trial

## INTRODUCTION

Sudden cardiac death (SCD) has become the leading cause of death in patients with left ventricular dysfunction^[1]^. A large number of randomized clinical trials (RCTs) have proved that implantable cardioverter-defibrillator (ICD) can terminate life-threatening ventricular arrhythmias effectively and reduce mortality significantly^[2,3]^. Therefore, ICD has become the first-line therapy for prevention of SCD for patients with heart failure and reduced left ventricular systolic function in the U.S. and European guidelines. ICD gained a class 1 recommendation^[4,5]^. However, the evidence in favor of ICD is much stronger for patients with ischemic heart disease than it is for patients with heart failure from other causes^[6]^. Over the past two decades, several RCTs concerning non-ischemic heart failure were carried out with controversial results^[6-9]^. The cardiomyopathy trial (CAT), which randomly assigned 104 patients with recent onset of dilated cardiomyopathy and an ejection fraction (≤30%) to receive ICD in combination with medical treatment or medical treatment only did not show lower mortality with ICD^[7]^. Desai et al.^[10]^ performed a meta-analysis of 7 RCTs as regards ICD in patients with non-ischemic heart failure and showed a significant 31% overall reduction in mortality with ICD therapy. More confusingly, the recent Danish Study to Assess the Efficacy of ICDs in Patients with non-ischemic Systolic Heart Failure on Mortality (DANISH), which randomized 1,112 patients with symptomatic systolic heart failure [ejection fraction (EF) 35%] to ICD in combination with optimal medical treatment or optimal medical treatment only, did not provided evidence in favor of ICD implantation^[6]^. Given the confusing situation of ICD application in non-ischemic heart failure, we performed an updated system review and meta-analysis.

## METHODS

### Search Strategy and Selection Criteria

We systematically reviewed relevant studies between January 1, 1966, and August 31, 2016, by searching Embase, PubMed, and the Cochrane Central Register of Controlled Trials. We used the terms implantable cardioverter-defibrillator, implantable defibrillator, randomized controlled trial, and clinical trial to identify RCTs. We considered all potentially eligible studies for review, regardless of the primary outcome or language. We also performed a manual search, by searching the reference lists of key studies.

### Inclusion Criterion and Data Abstraction

We regarded studies as eligible for inclusion if they met the following criteria: the study design was a prospective RCTs; the study population was non-ischemic heart failure with high risk of SCD including symptomatic or asymptomatic ventricular tachyarrhythmia or those with depressed left ventricular ejection fraction (LVEF), patients were randomly assigned to ICD in combination with medical therapy or medical therapy only; and the main endpoints included all-cause mortality. If the study included patients with cardiac resynchronization therapy (CRT) or cardiac resynchronization therapy- cardioverter -defibrillator (CRTD), the proportion of patients with CRT or CRTD should be matched between groups to eliminate the bias caused by CRT. Trials are excluded if they contained survivors of SCD or unstable ventricular arrhythmias. Trials which studied heart failure because of coronary artery disease are also excluded.

Two investigators (L Tang and Zw Zhu) independently reviewed the articles following the inclusion and exclusion criteria and assessed relevance of the articles. Disagreements were resolved by discussion or consultation with a third investigator (Xq Hu). The following data were abstracted from the selected articles: total number of participants, inclusion criterion, study design, age, sex, LVEF, New York Heart Association (NYHA) class, ICD type, duration of follow-up, all-cause mortality and cardiac mortality.

### Data Analysis

Meta-analysis was performed to calculate the risk ratio (RR) and 95% confidence interval (CI) of all-cause mortality. Statistical heterogeneity among the trial-specific RRs was checked and quantified by the I^2^ statistic, and a *P*-value ≤0.05 was considered statistically significant. When no significant statistical heterogeneity was identified, the fixed effect was preferentially used; otherwise, a random-effects model was used as an alternative. Data analysis will be performed on an intention-to-treat basis. All analyses were performed using Review Manger Software, RevMan 5.3.

## RESULTS

### Search Results

**Fig. 1:**
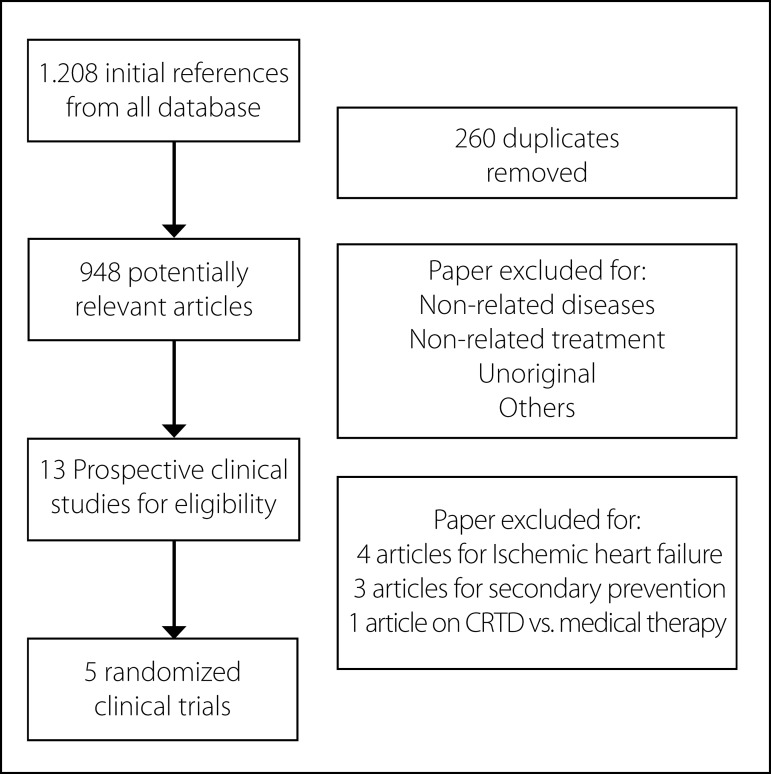
Flow diagram of literature searched for these reviews. CRTD = Cardiac resynchronization therapy-cardioverter-defibrillator

**Fig. 2:**
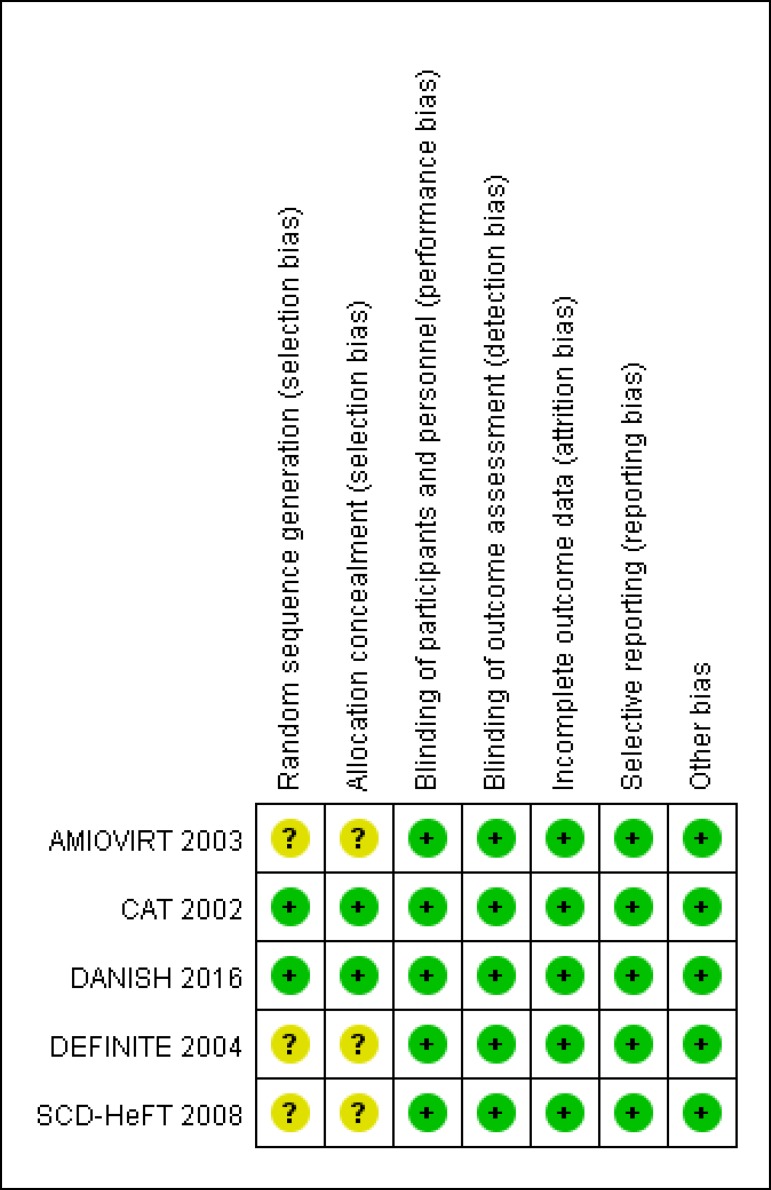
Bias assessment using Cochrane Collaboration tool.

The combined search strategy identified 1,208 potential relevant manuscripts. On the basis of the abstract evaluation, 13 of these studies were considered potentially eligible for inclusion and their full-texts were analyzed (Figure 1). We excluded seven, four of them studied the effectiveness of ICD in patients with ischemic heart failure, and three were on the secondary prevention of ICD in patients with SCD. Cochrane Collaboration's tool was used to assess risk of bias^[11]^. After quality assessment, five high-quality trials were eligible for further pooling analysis (Figure 2). The main features of the five included studies have been presented in Table 1.

**Table 1:** Main features of included articles.

Study	Inclusion criteria	Study design	Patients	ICD	Type of ICD	Follow-up (m)	Intention-to-treat	Controlled 1-y mortality (%)	Main result (RR reduction)
CAT	EF≤0.35; NYHA II-III; DCM	ICD *vs.* drugs	104	50	ICD	66±26	Yes	3.7	54%
AMIOVIRT	EF≤0.35; DCM; NYHA II-III; asymptomatic NSVT	ICD *vs. *amiodarone	103	51	ICD	24±16	Yes	10	No statistical significance
DEFINITE	EF≤0.35; DCM; NYHA I-III; NSVT	ICD *vs.* drugs	458	229	ICD	29±14	Yes	6.2	No statistical significance
SCD-HeFT	EF≤0.35; NYHA II-III	ICD *vs*. amiodarone *vs.* placebo	1676	829	ICD	45.5	Yes	7.2	31%
DINISH	EF≤0.35;NICM;NT-proBNP≥200pg/ml	ICD/CRTD *vs.* drug/CRT	1116	556	ICD/CRTD	68±19	Yes	3.2	No statistical significance

AMIOVIRT=Amiodarone vs. Implantable Defibrillator Randomized Trial; CAT=cardiomyopathy trial; CRTD=cardiac resynchronization therapy-cardioverter-defibrillator; DANISH=Danish Study; DCM=dilated cardiomyopathy; DEFINITE=Defibrillators in Non-Ischemic Cardiomyopathy Treatment Evaluation; LVEF=left ventricular ejection fraction; NICM=non-ischemic systolic heart failure; NYHA=New York Heart Association; NSVT=non-sustained ventricular tachycardia; SCD-HeFT=Sudden Cardiac Death in Heart Failure Trial

### Characteristics of Studies

The five primary prevention of non-ischemic heart clinical trials are the Cardiomyopathy Trial (CAT), the Amiodarone *versus* Implantable Defibrillator Randomized Trial (AMIOVIRT), the Defibrillators in Non-Ischemic Cardiomyopathy Treatment Evaluation (DEFINITE), the Sudden Cardiac Death in Heart Failure Trial (SCD-HeFT), and the Danish Study (DANISH). Substantial heterogeneity among studies was inevitable. The CAT, AMIOVIRT, and DEFINITE are all patients with non-ischemic cardiomyopathy^[7,8,12]^. However, the SCD-HeFT study also included ischemic cardiomyopathy^[9]^. Only patients with non-ischemic cardiomyopathy were included in the study. The DANISH trial randomized patients with non-ischemic cardiomyopathy to ICD/CRTD in combination with optimal medical treatment, or optimal drugs treatment/CRT^[6]^. Given the matching ratio of CRT or CRTD between ICD group and control group, this trial was included. One other thing to note was that the Comparison of Medical Therapy, Pacing, and Defibrillation in Heart Failure (COMPANION) was excluded, which randomly assigned patients with advanced heart failure to optimal pharmacologic therapy alone or in combination with CRTD^[12]^. The COMPANION trial might overstate the benefits of ICD in combination with function of resynchronized pacing, as CRT alone already has a benefit on survival^[13,14]^. Furthermore, patients with ischemic cardiomyopathy was also included, which made data extract impossible. Last but not least, any comparison of defibrillator with antiarrhythmic drugs reveals only the relative effect of these two therapies, not the difference between treatment and no treatment. The AMIOVIRT, SCD-HeFT both compared amiodarone with ICD which might lead to bias inevitably. Given no beneficial effect of amiodarone on survival, those studies were included^[1]^. Finally, our meta-analysis included 2,573 patients with non-ischemic heart failure randomized to ICD group or optimal pharmacologic therapy group (Table 2).

**Table 2:** Baseline clinical characteristics of patients.

Study	Age (y)	Male (%)	EF (%)	No-ischemic (%)	NYHA (%)		Pharmacological therapy (%)			
					II	III	ACEI/ARB	β-blocker	Amiodarone	Digoxin
CAT	52±11	83	24	100	67	33	94	4	NR	86
AMIOVIRT	59±11	72	23	100	35	25	85	52	50	71
DEFINITE	58	71	21	100	54	21	97	86	4	42
SCD-HeFT	60	77	25	47.3	71	29	NR	69	NR	67
DNISH	64±8	72	25	100	54	45	97	92	6	NR

ACE/ARB=angiotensin-converting enzyme inhibitor/angiotensin receptor blocker; AMIOVIRT=Amiodarone vs. Implantable Defibrillator Randomized Trial; CAT=cardiomyopathy Trial; DANISH=Danish Study; DEFINITE=Defibrillators in non-Ischemic cardiomyopathy treatment evaluation; LVEF=left ventricular ejection fraction; NYHA=New York Heart Association; SCD-HeFT=Sudden Cardiac Death in Heart Failure Trial

### All-Cause Mortality

**Fig. 3:**
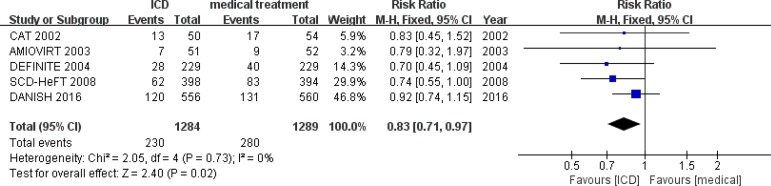
All-cause mortality among patients with non-ischemic heart disease randomized to implantable cardioverter -defibrillator (ICD) vs. medical treatment only in primary prevention.

The CAT, SCD-HeFT trials showed significant reduction in all-cause mortality with RR reduction ranging from 31%-54%^[7,9]^. However, the AMIOVIRT, DEFINITE, DANISH trials have not shown a statistical reduction in all caused mortality^[6,8,12]^. When the results of five randomized clinical trials were pooled, no statistical evidence was found on the pooled evidence of heterogeneity (I^2^=0, *P*=0.77). Pooled analysis using a fixed-effects model showed the summary RR for all-cause mortality was 0.83 (95%CI: 0.65-0.96, *P*=0.02) (Figure 3).

### SCD

**Fig. 4:**
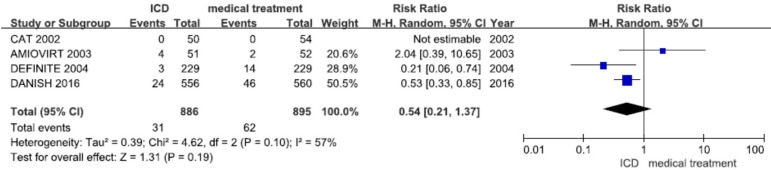
SCD among patients with non-ischemic heart disease randomized to ICD vs. medical treatment only in primary prevention.

The CAT and AMIOVIRT trails have not shown significant reduction in SCD. However, a tendency towards a reduction in SCD by ICD therapy was found in the DEFINITE and DANISH trials (RR: 0.2, CI: 0.06-0.71; RR: 0.50, CI: 0.31-0.82, respectively). Only a substudy of the SCD-HeFT trial was included and we could not extract the exact number of SCD in patients with non-ischemic heart failure. This study was included when we calculated the pooled effects of SCD. Moderate heterogeneity was found (I^2^=57%, *P*=0.1). Pooled analysis using a random-effects model have not shown reduction in SCD (RR: 0.54, CI: 0.21-1.37) (Figure 4).

## DISCUSSION

Our updated meta-analysis showed that, compared with optimized medical treatment, ICD in combination with medical treatment can yield improved outcome in patients with non-ischemic heart failure. In order not to overstate the benefits of ICD, the COMPANION study was excluded which randomized patients to optimal medical treatment in combination with CRTD or optimal medical treatment only. This analysis was robust in sensitivity. Finally, it is important to notice that the benefit of ICD is less compared with previous meta-analysis because of inclusion of the recent DANISH study^[9,10]^.

Over the past two decades, ICD implantation in patients with ischemic heart failure has been associated with improved outcome. Theuns et al.^[15]^ performed a meta-analysis as regards ICD in patients with ischemic heart disease. Pooled analysis showed a 29% RR reduction in all-cause mortality. However, the effectiveness of ICD in patients with non-ischemic heart failure is controversial. Meta-analysis of ICD secondary prevention trials have not shown more benefits compared with medical treatment only^[10]^. When new evidence occurs, we performed an updated meta-analysis, and showed that ICD therapy in combination with medical treatment improved outcome of patients with non-ischemic heart failure.

Our meta-analysis must be viewed in the context of its limitation. The treatment of heart failure has improved greatly with the implication of CRT, beta-blocker, and mineralocorticoid-receptor antagonist. Among this five RCTs, only CAT and SCD-HeFT were in favor of ICD implantation. The CAT study was not important in the pooling analysis because of its small sample size. The SCD-HeFT enrolled patients between 1997 and 2001, beta-blocker and mineralocorticoid-receptor antagonist were not well managed among patients at that time. Furthermore, the differentiation between ischemic heart and non-ischemic heart failure was mainly based on patient history, which was quite inaccurate compared with coronary angiography or computed tomographic (CT) angiogram used by the DANISH study. As we known, the evidence for a benefit of ICD is much stronger for patients with ischemic heart failure. The SCD-HeFT might overstate the benefits of ICD in patients with no-ischemic heart failure. The DANISH study of which more patients accepted ACEI/ARB, beta-blocker, mineralocorticoid-receptor antagonist, and CRT showed lowest mortality rate.

With optimized medical treatment in combined with CRT, ICD implantation in patients with non-ischemic heart failure has not brought further benefits.

Second, given the small number of studies included, publication bias in favor of ICD therapy cannot be inevitable. Although an extensive search strategy was performed, some studies might not be included in this meta-analysis.

## CONCLUSION

Although our finding lend support to the use of ICD in combination with optimal medical treatment improves the outcome of non-ischemic heart failure, further studies are needed to establish the optimal approach to treatment of non-ischemic heart failure.

**Table:** 

Authors' roles & responsibilities	
ZX	Conception, acquisition, analysis, interpretation of data, work review; final approval of the version to be published
LT	Conception, acquisition, analysis, interpretation of data, work review; final approval of the version to be published
CC	Conception, acquisition, analysis, interpretation of data, work review; final approval of the version to be published
JH	Conception, acquisition, analysis, interpretation of data, work review; final approval of the version to be published
ZZ	Conception, acquisition, analysis, interpretation of data, work review; final approval of the version to be published
XH	Conception, acquisition, analysis, interpretation of data, work review; final approval of the version to be published
